# London Temperance Hospital

**Published:** 1902-04-05

**Authors:** 


### LONDON TEMPERANCE HOSPITAL.

### SPEECH BY THE LORD CHIEF JUSTICE.

At the London Temperance Hospital it is the custom to follow the annual court of Governors by a visit to the wards, and then by a public gathering. At this year's meetings, held on the 24th ult., the attendance was less than usual owing to the inclement weather, but even cold wind and pelting rain did not prevent the public function in the evening, at which the Lord Chief Justice presided, from being a numerously attended, an interested, and an enthusiastic gathering, a large proportion of those present being ladies.

Mr. Alderman Strong, the Chairman of the Board of Management, presided at the routine meeting in the afternoon, when the proceedings, consisting of the adoption of the report, the election of officers, and other more or less formal matters, were run through with business-like celerity and precision. The board in their report referred first to the great regret felt at the death of Mr. Thomas Cash, who, in succession to the late Duke of Westminster, was elected President of the institution in March 1900, but died in September 1901. Regret was also expressed at the death of the Hon. Conrad Dillon. The report went on to state that the increasing work of the surgical department led in the May of last year to the appointment of Mr. H. J. Paterson, F.R.C.S., as assistant surgeon to Dr. Collins. The pressure upon the resident medical staff had made desirable the appointment of Dr. Burrows as assistant in the out-patient and casualty departments. Miss Lucas, whose resignation had been accepted with great reluctance, had been succeeded as matron by Miss Isabel Richardson. The in-patients admitted in 1901 were 1,299, of whom 877 were dismissed cured, and 237 relieved. The deaths were 107, or 8·2 per cent. In 1900 the admissions were 1,282, the cures 851, and the deaths 117 or 9·1 per cent. Of the cases admitted in 1901, there were 34 of a moribund character, and but for those the rate of mortality would have been but 5·6 per cent. Since the opening of the hospital in 1873 the in-patients, down to December 31st, 1901, numbered 19,209, of whom 11,249 were dismissed cured. The deaths were 1,397, a death-rate of 7·2 per cent. The out-patients were 9,007 in 1901, as compared with 8,327 in 1900. The visits in the respective years were 23,534 and 21,015, an increase of 2,519 in 1901. In the casualty department, 12,846 patients were treated in 1901, as compared with 14,012 in 1900. The visits were respectively 33,951 and 32,361, an increase of 1,590 in 1901.

The Temperance Hospital's Special Status.

In concluding their report the Board state: We are exceedingly anxious, as a stimulus to zeal and liberality, that our own friends, and the public at large, should realise the unique position of this institution, as the only general hospital which devotes itself to the systematic investigation of the treatment of medical and surgical cases without the ordinary use of alcohol. We recognise, and rejoice in, the diminished use of alcohol in most of the general hospitals of London, but, up to the present time, the Temperance Hospital is the only one which not only excludes alcoholic beverages from the dietary, but which also conducts the non-alcoholic treatment of disease under constant scientific observation. The potency of alcohol as a drug plainly calls for special caution in its medicinal exhibition; and as it is the physical cause of drunkenness in all its degrees, and with all its evils, no friend of science, or lover of humanity, can desire its administration in times of weakness and sickness, except when it is deemed necessary for the recovery of patients. To prosecute this inquiry is the special work of our hospital, and that it has been pursued with success may be inferred from the fact that out of 19,209 in-patients treated in 28 years, the visiting staff has not considered it needful to prescribe alcohol, as a drug, in more than 52 cases, the recoveries being 20 and the deaths 32 following its use. The practice of this hospital in other respects, and in the admission of the worst cases of all kinds, has been that of other general hospitals, and the death-rate over the whole period has been only 7·2, a result which places the Temperance Hospital on, at least, a level with similar institutions. Considering, therefore, the specific investigation carried on exclusively in this hospital, we have reason to appeal for support to the scientist, the economist, and the philanthropist. Those who consider our experience satisfactory should assist in providing means for its confirmatory investigation, and those who are not yet prepared to regard it as complete may properly be urged to help in its continuance. On the determination of the question at issue great reforms and consequences depend, and in that determination the work of this hospital is, we believe, destined by Divine Providence to form an essential and powerful factor.

### Lord Alverstone's Address.

At the well-attended evening meeting, the Lord Chief Justice (Lord Alverstone) presided, being supported on the platform by Mr. Alderman Strong, Dr. Dawson Burns, Colonel Sheffield, and others. The proceedings were opened with prayer. Lord Alverstone, who had spent some time in going through the wards of the hospital, said that when he entered the building he was met by a tablet which took his memory back to one of the first workers in the cause of temperance—Dr. Benjamin Ward Richardson, than whom no one brought to bear on medical and scientific questions a more open mind, or who pursued them more relentlessly with a determination to get at the truth. There were other distinguished supporters of that hospital in all walks of life: Mr. Cash, whose loss they were deploring; Mr. Sturge, in whose munificence they were rejoicing. In the army there was Lord Roberts, whose example and teaching had done so much; in the navy, Sir William White, the chief constructor; and no one who knew Miss Weston's work at Portsmouth could doubt what temperance had done for the navy. The late Duke of Westminster, too, had always been a warm supporter of that hospital. He himself was glad to say a word in praise with regard to the work from a lawyer's point of view. For the past 30 years he had advocated temperance in season and out of season; in politics and out of politics. For many years he had been closely connected with the Church of England Temperance Society, and he was glad to have been permitted to speak, not infrequently, on behalf of that society, and he supported that hospital because it was the embodiment of the principle he had always upheld, that the backbone of the temperance movement was that people could not be driven into it, but could be led. That institution was a powerful agency in leading public opinion and would be so long as the walls stood, and the work done drew an ever-increasing number of patients within them.

### The Progress of Medical Opinion.

If, 28 years ago, they had canvassed the hundreds of medical men then practising in London, and the larger number of those residing in various parts of England, and asked them if it were possible to conduct a general hospital on temperance lines, they would have replied that it was impossible. But for 28 years the London Temperance Hospital had existed, and treated nearly 20,000 patients, and in only 52 cases had it been found necessary to use alcohol. This was a fact which 28 years ago would not have been believed, and was a testimony which greatly redounded to the honour of the work carried on within the walls of the institution. (Applause.) The patients sent out were willing witnesses to the advantage of non-alcoholic treatment. Their medical men and their nurses were also witnesses to the beneficial effects of non-alcoholic treatment. The results attained had been followed in every hospital, and were leading to the reduction of the use of alcohol in them. In the few cases treated at that hospital with alcohol, what was administered was rectified spirits of wine, which no one would take for the pleasure of taking it. Intemperance, every judge in the land would tell them, was at the bottom of more than 50 per cent. of the crime of the country. If they could put a stop to intemperance and gambling their prisons would soon be empty. (Applause.) He felt the greatest interest in the London Temperance Hospital, and was confident it would do immense good. With great deference, as a layman, he would suggest that it would be of the greatest value, from a statistical point of view, if the Board of Management would furnish a clear distinction in their future reports as to the number of abstainers and non-abstainers amongst adults treated in the hospital as distinct from the children. His presence there would show that he felt the greatest interest in that institution, and regarded it as a successful experiment of the most valuable kind.

His lordship was followed by Dr. Collins, L.C.C., Dr. Addinsell, Alderman Strong, Mr. Revell, ex-Mayor of Windsor, and others, the speeches being followed throughout with the liveliest interest, and punctuated with frequent and enthusiastic applause.

